# Horizontal gene transfer dynamics and distribution of fitness effects during microbial *in silico *evolution

**DOI:** 10.1186/1471-2105-13-S10-S13

**Published:** 2012-06-25

**Authors:** Vadim Mozhayskiy, Ilias Tagkopoulos

**Affiliations:** 1 Department of Computer Science and UC Davis Genome Center, University of California Davis, Davis, California, 95616, USA

## Abstract

**Background:**

Horizontal gene transfer (HGT) is a process that facilitates the transfer of genetic material between organisms that are not directly related, and thus can affect both the rate of evolution and emergence of traits. Recent phylogenetic studies reveal HGT events are likely ubiquitous in the Tree of Life. However, our knowledge of HGT's role in evolution and biological organization is very limited, mainly due to the lack of ancestral evolutionary signatures and the difficulty to observe complex evolutionary dynamics in a laboratory setting. Here, we utilize a multi-scale microbial evolution model to comprehensively study the effect of HGT on the evolution of complex traits and organization of gene regulatory networks.

**Results:**

Large-scale simulations reveal a distinct signature of the Distribution of Fitness Effect (DFE) for HGT events: during evolution, while mutation fitness effects become more negative and neutral, HGT events result in a balanced effect distribution. In either case, lethal events are significantly decreased during evolution (33.0% to 3.2%), a clear indication of mutational robustness. Interestingly, evolution was accelerated when populations were exposed to correlated environments of increasing complexity, especially in the presence of HGT, a phenomenon that warrants further investigation. High HGT rates were found to be disruptive, while the average transferred fragment size was linked to functional module size in the underlying biological network. Network analysis reveals that HGT results in larger regulatory networks, but with the same sparsity level as those evolved in its absence. Observed phenotypic variability and co-existing solutions were traced to individual gain/loss of function events, while subsequent re-wiring after fragment integration was necessary for complex traits to emerge.

## Background

Horizontal Gene Transfer (HGT) is the transport of genetic material within and across species. It is a mechanism of genetic exchange complementary to vertical transfer, which occurs through cell division and results in the transfer of genetic information from an ancestor to its offspring cells. Although largely ignored in the past, recent phylogenetic evidence suggests that its impact on bacterial evolution is significant and should be investigated more thoroughly [1,2]. For instance, it has been estimated that up to a 32% of the bacterial genome is acquired by HGT [3]. However, even this number is a lower bound of the HGT events that take place through bacterial evolution, since only a small fraction of transferred material is positively selected, fixed, and consequently, observable through phylogenetic analysis [4]. The current belief is that fixation is more probable for auxiliary genes which encode specific functions [5], and that horizontally transferred genes are integrated at the periphery of the network while core network parts remain evolutionarily stable [6].

Due to our limited ability to observe HGT dynamics in an experimental setting, theoretical models have been traditionally employed to elucidate the impact of HGT on evolution. Continuous [7,8] and stochastic [9-11] models were developed to analyze the interplay between rates of HGT and selection pressure parameters. In these models, organisms are often viewed as having only two states, depending on whether they carry a specific allele [9]. As such, these models may provide an insight into the fixation dynamics for different alleles, but cannot describe the emergence of new functions and evolution of the regulatory networks after gene transfer. Kinetic models [7,11] are used to study the short-term dynamics of the vertical and the horizontal "flow" of genes between the organisms, but since they ignore selection pressure, they cannot properly describe the effect of the horizontal gene transfer on evolution. Furthermore, it was theoretically shown that transferred genes can be successfully fixed in a population when the HGT rate is comparable to the mutation inactivation rate [9], and a simple population model was used to show that high rates of HGT may affect the evolution rate [10].

**Figure 1:**
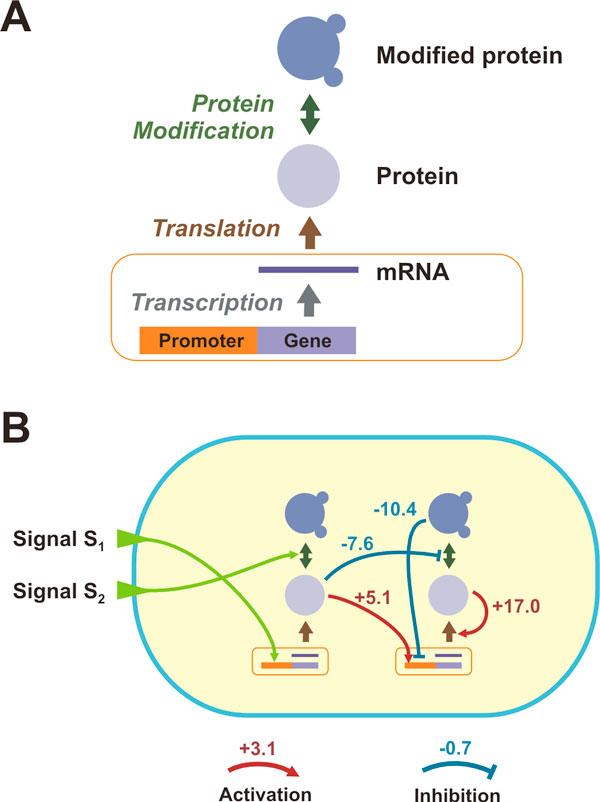
**Basic cellular modeling in our simulation framework**. (**A**) A "triplet": capturing the processes of transcription, translation, and post-translational modification. (**B**) Example of a gene regulatory and biochemical network in an organism where environmental signals (e.g. oxygen, temperature, etc.) regulate the expression of certain genes/proteins. The value at each node of the graph corresponds to the number of molecules of a given molecular species. Red/blue arrows denote positive/negative regulation and their corresponding weights.

A general problem in the studies that combine HGT and evolution is that selection and fitness have been modeled as an arbitrarily-assigned function of allelic or genotypic frequency, without the presence of an integrated gene regulatory or environmental model. Previous models, although insightful, have a limited scope as they lack any notion of gene regulation, cellular networks and processes, multi-scale structures, and temporal expression dynamics. As such, the distribution of fitness effects for HGT, and its impact to the organization, topology, and kinetics of the underlying biological networks was never investigated until now. To address this questions, we extended our previous work [12] to develop a multi-scale simulation framework that is capable of simulating the evolution of unicellular organisms in the presence of HGT. As in the original model, each *in silico *organism encompass functions and parameters that model basic biological phenomena, while its core consists of a gene regulatory and biochemical network with abstract molecular representations. Instead of imposing an arbitrary, artificial selection function (correlation, calculation, etc.) as in previous studies, we instead created an environmental model that includes nutrients. The model has been extended to incorporate the Horizontal Gene Transfer in addition to other cellular (transcription, translation, modification, growth, and death) and evolutionary (mutation and natural selection) processes previously included in the model (see Methods and [13]). Additionally, to achieve higher population sizes, we extended the hybrid openMP/MPI model [14] to handle HGT events through message passing communication between cells that run in different processors. In a simulation, a fixed-size population of cells mutates, competes and evolves in well-defined, temporal, multivariate environments. Each cell comprises three types of nodes: Gene/mRNA, Protein, and Modified Protein (Figure 1A). The Promoter/Gene/RNA node captures gene regulation and transcription, while the Protein and Modified Protein nodes capture translation and post-translational modification (acetylation, phosphorylation, etc.), respectively. A "triplet" consists of a specific gene node and its products, i.e. the corresponding protein and modified protein node, and generally captures the "central dogma" of molecular biology (Figure 1A). Each organism has its own distinct gene regulatory and biochemical network (i.e. a collection of various triplets and weighted regulatory edges) that can be depicted as a directed weighted graph (see Figure 1B). There exists a set of "special triplets", which are common in all cells, and encode physiological responses. Mutations can change any node or link parameter, and triplet duplications or deletions, allow the network to grow or shrink in size, respectively. It is important to note that we do not impose any objective function or arbitrary selection. Instead, we model the environment in which synthetic organisms live and evolve, which consists of signals, nutrients and other chemicals (e.g. toxic compounds), with concentrations that can fluctuate over time. In this work, every environment has only one nutrient type and each organism possesses one special triplet, T0, whose expression allows the organism to metabolize the nutrients that are present. Since nutrients are present for a short duration, organisms that evolve the capacity to infer their presence and be prepared (e.g. express the metabolic triplet) have a selective advantage, in analogy to real microbial systems. We utilize this framework to address questions regarding the impact of HGT on trait evolution, fixation, and gene regulatory network organization.

**Figure 2:**
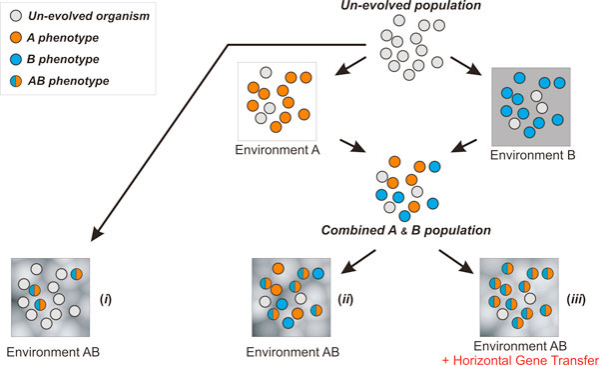
**General overview of the simulated ecological setting**. Microbial population evolves under a complex environment AB ("single-step" adaptation) either directly (***i***), or through a first step of evolution in less convoluted, but still related, environments A and B with (***ii***) or without (***iii***) the presence of HGT events.

An overview of the simulation setting discussed in this paper is illustrated in Figure 2. We start with a random initial population of cells and three dynamic environments, namely A, B and AB, where the latter is the combination of the first two and of higher complexity. The un-evolved initial population is either placed directly into environment AB, or it is first evolved in the intermediate environments A and B, which leads to two distinct populations. These two populations are subsequently randomly sampled (keeping the same effective size) to form a final population that is then placed in the environment AB with and without HGT. This setting allows us to introduce complementary, but sub-optimal, phenotypes (as it is the case of clonal interference in homogenous populations), and to address questions regarding evolution in correlated environments of increasing complexity.

**Figure 3:**
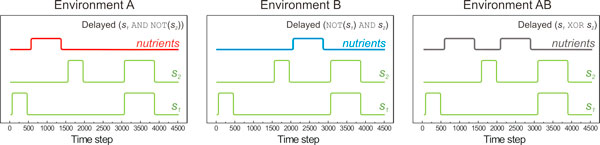
**Environmental signals and nutrient abundance**. Environmental signals (green) and nutrient abundance (red, blue and grey) for the three environments (A, B, and AB (XOR) respectively) shown as a function of time steps within one epoch. Nutrient presence is a delayed function of the two signals. The same signals and nutrients are present in all environments (i.e. all cells have the same special triplet function), but with different temporal dynamics. One epoch is shown in each plot, which consists of 4,500 time units total.

In the setting discussed here, two signals *s_1 _*and *s_2 _*carry information regarding the presence of nutrients in the environment (Figure 3). The input/output correlation of the AB environment is a delayed XOR: *Nutrients Presence [XOR] = Delayed *(*s_1 _*XOR *s_2_*). Similarly, the correlation-structure of environments A and B is *Nutrients Presence [A] = Delayed *(*s_1 _*AND NOT(*s_2_*)) and *Nutrients Presence [B] = Delayed *(NOT(*s_1_*) AND *s_2_*), respectively. The introduction of a delay in the signal/nutrient correlation further increases the evolutionary complexity of the environment, as organisms now have to account for it, through the underlying network topology and dynamics. In the absence of delays between nutrient-signal occurrences, we obtained the same observations, but evolution took place faster, and networks tended to be less complex. The three environments were not randomly selected: despite the fact that the combined AB environment (delayed XOR) is a simple combination of the A and B environments, its complexity is significantly higher when compared to the other two (A and B). The main reason behind the increased complexity of environment AB is the fact that is not linearly separable [15], in contrast to both A and B environments that can be separated linearly. As such, a solution is easier to evolve in the latter, which is also evident by the corresponding evolution rates (Table 1). To assess the fitness level of each organism, we report the Pearson correlation between nutrient abundance and response protein (T0 triplet) expression level over a predefined interval of time, which we call an "epoch" (4,500 time units in our simulations). We stress that this similarity measure is used for visualization purposes as a proxy to each organism's fitness, and at no point participates or interferes with the selection or evolutionary trajectory of cells during the simulation. High correlation between nutrients and response protein concentration implies an efficient mechanism to metabolize nutrients, as activation of the costly metabolic pathway takes place only when needed.

**Table 1:** Rate of adaptation to single environments A and B, and a complex XOR environment in different experimental scenarios

	Emergence of the organism with fitness *w*			
	*w *> 0.75		***w ***>**0.90**	
	
	Success rate	Average speed, *epochs*	Success rate	Average speed, *epochs*
Un-evolved → *XOR^a^*	18/32	2485	15/32	2489
Un-evolved → *A^a^*	30/32	1043	29/32	1067
Un-evolved → *B^a^*	31/32	1217	31/32	1319
{*A *&*B*} → *XOR^b^*	58/64	234	47/64	448
{*A *&*B*} → *XOR ***+ HGT **^*b*^	64/64	138	48/64	406
Acceleration of adaptation by HGT	1.7		1.1	

The probability and the speed of *XOR *phenotype emergence are shown for two fitness thresholds 0.75 (evolved organism) and 0.90 (refined evolved organism). Probability is calculated as the ratio between the number of experiments with the maximum fitness above the threshold in the end of the run, to the total number of experiments. Average speed is the average epoch number at which maximum fitness surpasses the threshold (the speed calculated only for successful experiments, therefore it is overestimated for cases with a low adaptation probability).

*^a ^*32 experiments; 4,000 epochs each.

*^b ^*64 experiments; 2,000 epochs each.

## Methods

### Simulation framework

A population of organisms in our simulations is composed of a fixed number of organisms with the distinct gene regulatory and biochemical networks. Networks are composed of "triplets" (Figure 1A): mRNA, protein and modified protein nodes. Each node can associate and regulate any other node (although frequencies of certain interactions can be restricted), which represents regulation of translation, transcription and modification of the nodes within the network. Kinetics of expression is parameterized by several continuous variables which map to biological properties such as basal expression, degradation probability and regulatory strength. Overall, the network of each organism is represented as a weighted directed graph, with weights describing the strength of regulation (activation or inhibition) and node values being the number of molecules of each particular type. Node values are updated at each time step of simulation according to the expression model described in the next section in more details.

**Figure 4:**
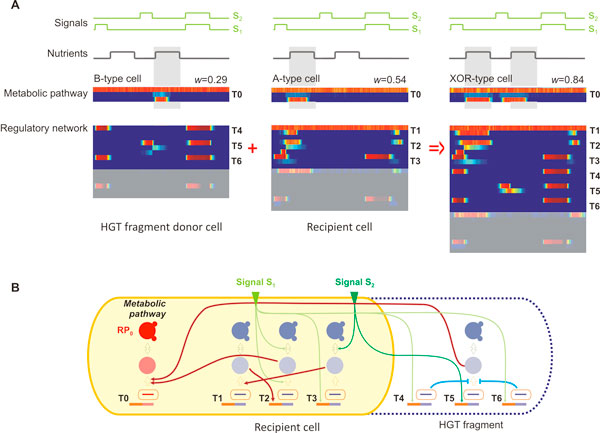
**Combined *XOR *phenotype is formed by HGT between cells evolved in single *A *and *B *environments**. (**A**) Microarray-like expression levels with one epoch (4500 time steps) for three cells from left to right: donor cell for the HGT transfer evolved in the *B *environment (highlighted triplets T0, T4-T6 form a minimal network), recipient cell evolved in the *A *environment (triplets T0-T3 is the minimal network), and a final combined cell with a minimal network T0-T6. Shaded expression levels are for triplets outside the minimal functional network. Environmental signals and the nutrient abundance as a function of time are shown at the top in green and grey, respectively. The Pearson correlation between expression levels of the modified protein from the metabolic pathway RP_0 _(bottom row in metabolic pathway T0) and nutrient abundance is the fitness of the cell *w*. (**B**) Corresponding gene regulatory and biochemical networks (only minimal networks are shown). Network for cell of type *A *(recipient cell) is shown on the solid yellow background. Triplets transferred in HGT fragment from the cell of type *B *are enclosed in the dashed segment on the right. Each triplet (T1-T6, and the metabolic triplet T0 on the left) consists of three nodes from bottom to top: mRNA, protein, and modified protein. Red and blue arrows show activation and inhibition (strength of regulation is not shown). Regulation by two external signals S_1 _and S_2 _is shown with green arrows.

Two environmental signals vary over a period of 4,500 time steps, which is the length of one epoch (Figure 3). Full simulation length of simulations was 2,000 to 4,000 epochs depending on the speed of adaptation to a particular environment. All organisms in the population are regulated by (i.e. "connected to") the environmental signals in a probabilistic way: some nodes in the regulatory network can be regulated by one of the external signals, rather than by the nodes within the network. In addition to its regulatory network, each organism has a unique metabolic pathway (represented as the "RP_0_" pathway of triplet T0, see for example Figure 4B) which, when expressed, can metabolize available resources in the environment.

Nutrients availability correlates with the environmental signals (e.g. a delayed XOR function in Figure 3). Organisms cannot directly sense the presence of resources; however they can potentially infer their future presence, if they are evolved to process information from various environmental signals through biochemical and regulatory interactions. Energy can be acquired by the organism at any time point if both conditions are satisfied: *(i) *nutrients are present in the environment, and *(ii) *the response pathway (RP_0_), which is part of the metabolic pathway, is being expressed. The high cost of metabolic protein production favors organisms that can time the production of RP_0 _with the presence of nutrients in the environment. Expression of the molecules and mutation events have an energy cost, as does the maintenance of molecular species (nodes). Costs per one time step of RP_0 _and any other node maintenance is 1 and 10 energy units respectively; energy gain per expressed RP_0 _per time step when nutrients are present is 50 energy units.

Once an organism reaches a certain energy level (1,600,000 energy units), it undergoes a division, increasing its genotype representation in the population, while its progeny replaces an existing organism with the low energy so that the fixed size of the population is preserved (probability of an organism being replaced is inversely proportional to its energy level). To assess the level of fitness of each organism, we report the Pearson correlation between nutrient abundance and RP_0 _expression level over one epoch, a predefined interval of time (4,500 time units in our simulations). We use fitness only to screen the degree of adaptation and fitness is not a parameter of the simulation. Fitness correlates with the division rates, since fitness is a measure of the organism's efficiency of metabolizing the nutrients.

Mutation events (*e.g*. transcription rate changes, node duplications, node deletions, signal connection etc.) occur stochastically at any time point and on any node, thus changing its internal network and potentially its phenotype, which in this context is synonymous to the regulatory and metabolic pathway expression.

### Mutation rates

Mutation rates in wild strain *E. coli *are estimated to be 2·10^-8 ^per generation, per base pair. DNA repair drops this by another 10^-2 ^[16], which results in 2·10^-10 ^error rate and consistent with other estimations [17,18]. With approximately 10^3 ^base pairs per gene in *E. coli*'s genome, the mutation rate per gene is about 3·10^-7^. However, a recent experimental study has found that these measurements are for large population sizes, where clonal interference plays a major role, and experimentation with population sizes closer to those encountered in our simulations (10^4^) showed that mutation rates are 1000 times higher than those measured previously [19]. In addition, in laboratory experiments under stress or with chemical mutagens, mutation rate increases another 10^2 ^up to 10^4 ^per gene, per generation. Similar results have been obtained in other microbes, such as yeast, where the mutation rate was estimated at 3·10^-10 ^per base pair, per generation, and therefore is about 10^-6 ^per gene [20]. To account for all these factors, we used here the following mutation rates: probabilities of weak (a change of a parameter within a Gaussian distribution of predefined width from the original value) and strong (a new value is drown for the parameter) mutations equal to 10^-5 ^and 4·10^-6 ^per triplet, per time step, respectively; probabilities of triplet creation and destruction are 10^-6 ^per triplet, per time step.

Although we used fixed size populations of 256 organisms in all experiments, evolution rate (measured as a slope of the maximum fitness function of time) scales linearly up to populations of at least 4096 cells as it is shown in Additional file 1. Evolution rate does not slow down below the linear dependence for larger populations: mutation events are still rare and sequential mutations do not occur before the previous ones are fixed. At the lower limit of plotted population sizes scaling is also linear, which suggests that genetic drift and random fixation of deleterious mutations dos not interfere with the evolution (in populations smaller than 32 organisms genetic drift become a major obstacle for adaptation). Therefore we assume that in our model neither clonal interference nor genetic drift is the major driving force of the evolution for the populations in the range of 256 to 4096 organisms, and the lower number was used to minimize the computational cost of simulations.

### Expression model

The expression model is described in detail in [12]. Briefly, the probability of molecule creation at each node and at each time step is a function of the regulatory effect of other nodes on that specific node, and the availability of substrate molecules. We model the molecule production probability as a two-level sigmoid function that captures activation thresholds and saturation effects for any given regulator and for the expression of any given node. As such, the molecule production probability of node *i *is given by:

$$G_{i}=basal_{i}+\left(1-basal_{i}\right)\cdot \tanh\left(\frac{\sum_{j=1}^{n}\left(w_{ij}\cdot f_{ij}\left(v_{j},{\tilde{m}}_{ij},{\tilde{s}}_{ij}\right)\right)-m_{i}}{s_{i}}\right)$$

where the sigmoid function *f_ij _*describes the regulatory effect of node *j *on node *i*:

$$f_{ij}\left(v_{j},{\tilde{m}}_{ij},{\tilde{s}}_{ij}\right)=\frac{1}{2}\cdot \left[1+\tanh\left(\frac{v_{j}-{\tilde{m}}_{ij}}{{\tilde{s}}_{ij}}\right)\right]$$

where *w_ij _*is the regulatory matrix element (i.e. the strength and direction that exerts node *j *to node *i*), *v_j _*is the value of node *j*, *m_i _*and *s_i _*the midpoint and slope of the target-specific sigmoid function, ${\tilde{m}}_{ij}$and ${\tilde{s}}_{ij}$ the midpoint and slope of the regulator specific sigmoid function, *n *is number of regulating nodes, *basal_i _*is the basal expression parameter.

Initial unevolved populations are composed from randomly generated cells with the following parameters: *w_ij _*is drawn from the double sided power law distribution with 1.5 exponent (therefore 10% of weights have an absolute value > 4); ${\tilde{m}}_{ij}\in \left[0,10\right];$${\tilde{s}}_{ij}\in \left[1,4\right];$*m_i _*∈[-1, 1]; *s_i _*∈ [1-4]; *basal_i _*is ∈[0, 0.5]; initial sparsity of the regulatory network is (0, ..., 0.2); connection to the input signals of each additional node is proportional to exp(-*n*), where *n *is the number of nodes already regulated by input signals; initial energy of the organism 800,000 energy units; size of the initial random network can be up to 15 triplets.

### HGT model

There are three mechanisms for HGT by which bacteria can acquire external DNA: transformation, conjugation and transduction (e.g. review [21]), which we capture through a probabilistic pair-wise model, where an HGT event between any two organisms in the population, or one organism and a genomic "fragment" (e.g. naked DNA present in the solution) occurs with a fixed probability.

**Figure 5:**
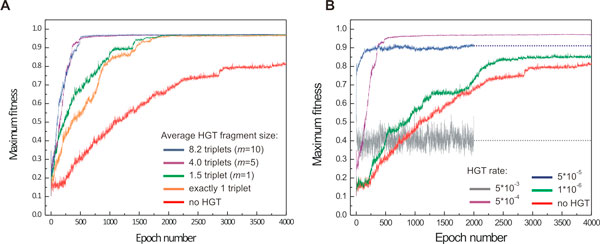
**Effect of HGT as a function of transferred fragment size and HGT rate**. (**A**) Emergence of fit phenotypes is accelerated with increasing average fragment length and saturates after the latter has reached the effective minimal network size; m denotes the middle-point of the fragment size probability density function (see methods), (**B**) evolutionary trajectories for different HGT rates, averaged over 32 simulations.

Experimentally observed HGT rates between bacteria in natural environments vary between 10^-7 ^and 10^-11 ^per generation, per cell [22-24], while in some cases the rate reaches 10^-3 ^to 10^-1 ^[24,25]. In our model a gene and its products are represented by triplets, and therefore HGT can be treated as inter-cellular transfer of one or more triplets. For every HGT event a random subset of triplets (sub-network) is copied from the donor cell and inserted into the regulatory network of the recipient cell. A model where the introduced genes had random dependencies yielded similar results. Original regulation of the metabolic pathway RP_0 _and triplet T0 by the transferred sub-network is preserved (Additional file 2). Parameter sweep for HGT frequency from fully evolved *XOR *networks to non-evolved organisms is shown in Figure 5. The observed optimal frequency of 5·10^-5 ^(per cell, per time step) of HGT events is in the upper range of the experimentally observed values, and consistent with the rest biological and evolutionary model.

Distribution of the fragment sizes in HGT events may vary greatly in the bacterial world and depends on the type of the transfer and experimental conditions. However in all three types of HGT the maximum size of the transferred DNA is limited by different parameters: in transduction by the capacity of the viral capsid, in conjugation by the time two organisms stay connected by a pilus, and in transformation by the stability of the naked DNA in the environment. In general the probability of transfer small fragments of meaningful DNA is higher than of larger ones. In our model triplets with preserved regulatory network are transferred from one organism to another, and the fragment size for an established HGT event is chosen using a probability density function as a normalized sigmoid function:

$$P\left(n\right)=\frac{1-\tanh\left(\frac{n-m}{s}\right)}{m\cdot \left(2+\ln\left(e^{-\frac{2m}{s}}+1\right)\right)}$$

where *n *is the fragment size in triplets, *m *and *s *are the middle point and slope of the probability density function, respectively; the denominator is a normalization coefficient. In most cases *s = m *was used throughout the paper, and therefore 67% of all transferred fragments were not larger than *m *triplets (Additional file 2). One exception is "exactly one triplet" plot in Figure 5B, which was obtained with a sharp step size distribution function so that exclusively single triplet fragments were transferred (*m *= 1.5 and *s *= 0.1). Unless otherwise mentioned, the default parameters for HGT in environments tested in our paper were set to (*s *= *m *= 5) which results in the expectation value for the size of HGT fragments equal to 4 triplets and slightly smaller than the average size of the minimal network (5 to 7 triplets, see Table 2).

**Table 2:** Complete and minimal network statistics for populations evolved in a XOR phenotype with and without HGT

	Full network				Minimal network			
	No HGT		HGT		No HGT		HGT	
Fitness *(St. Dev.)*	0.81	*(0.052)*	0.79	*(0.044)*	0.78	*(0.006)*	0.75	*(0.006)*
Triplets	8.8		13.8		5.5		6.7	
Links *(St. Dev.)*	335	*(157)*	338	*(136)*	10.6	*(0.03)*	14.1	*(0.03)*
Sparsity	0.39		0.22		0.11		0.10	
Modularity	3.8		10.1		3.3		3.1	

Statistics were calculated over 128 simulation runs, and in populations that evolved an *XOR *phenotype (environment AB) with and without HGT (2000 epochs). Only fit cells (fitness > 0.7) were included, resulting on an average 74% representation. Sparsity is calculated as the ratio of the number of edges to the maximum number of possible edges in a directed graph.

### Algorithm overview

The code is based on a stochastic simulation algorithm where mutation events occur randomly based on predefined probability distributions. We use an MPI model to distribute a population of cells to a set of computational nodes. Cells are distributed between MPI processes. Weak and strong scaling shows good scalability up to 1024 computational cores (NCSA Blue Print P5 system and preliminary tests on P7 drawer) with a near-linear speedup for a load of 8 cells per core. At every time step organisms mutate with predefined probabilities and node values are updated using the stochastic expression model described above; cells which exhausted their energy are removed from the population and replaced with new random cells (to start from a new point on the fitness landscape); cells which reach an energy above the division threshold are duplicated, and daughter cells replace cell with low energies to maintain a constant population size.

### Network reduction

To elucidate the *modus operandi *of each evolved network, which may have hundreds of nodes and links, we developed the following heuristic to reduce the network to its "minimal" form, in which only essential nodes and links remain. In this iterative procedure, the fitness effect of a link is assessed after its severance. The link is permanently removed if it is deemed non-essential (less than 5% fitness change). The procedure is repeated until the network cannot be reduced any further. Due to the stochastic nature of the expression model, fitness of a cell can vary as much as 30% between sequential epochs. For that reason the average fitness is evaluated over 10 epochs to reduce that variation to 2%. Multiples iterations over all edges with a tight removal threshold ensure gradual and stable reduction on the network to a near-optimal minimal sub-network.

### Distribution of fitness effect

Mean, variation, skewness and kurtosis for several experimentally measured DFEs are summarized in Table 3. Fitness effect (or selection coefficient) is usually defined as [*w(z_0_+dz)/w(z_0_)*]-1, where *w(z_0_) *and *w(z_0_+dz) *are the fitness of an organism before and after a mutation event, respectively. We use the same definition for the fitness effect of HGT events. Mean of DFE is the average deleterious effect of mutations or HGT; skewness is the measure of asymmetry (for a symmetric distribution it is zero and skewness is negative if the distribution is skewed to the left and *vice versa*); kurtosis is the measure of 'peakedness' (positive if distribution is 'sharper' then a Gaussian distribution).

**Table 3:** Proprieties of distribution of fitness effect

	Mean fitness change, %	Fitness variance	Skewness	Kurtosis	Percent of lethal events
**Mutations**					
Un-evolved populations	-6.6%	0.109	-0.014	3.10	33.0%
Evolved populations	-4.5%	0.032	-1.028	6.39	3.2%
**Horizontal gene transfer**					
Un-evolved populations	-5.1%	0.063	-0.129	4.49	14.4%
Evolved populations	-6.6%	0.048	-0.064	4.70	4.6%
**Experimental values (mutations)**					
VSV RNA virus^a^	-6.4%				26.4%
Bacteriophage f1^b,d^	-10.7%	0.037	-1.909	3.16	21.0%
Qβ bacteriophage^c,d^	-10.3%	0.018	-1.167	0.24	28.6%
*E. coli *(insertions)^e^	-3.2% to -1.2%	< 0.01			< 5%

DFE for mutation and HGT events in evolved and un-evolved populations. Calculated DFE and an overview of the experimental data for mutation events for comparison.

^a ^Single nucleotide substitutions in vesicular stomatitis virus [35].

^b ^Single nucleotide substitutions in Bacteriophage f1 [34].

^c ^Mutation accumulation experiment for Qβ bacteriophage [33].

^d ^Summary of results for mutation effects in RNA and single stranded DNA viruses [36].

^e ^Mini-Tn10 transposon insertions in *E. coli *[31].

## Results

### Effect of HGT on evolution in single and combined populations

**Figure 6:**
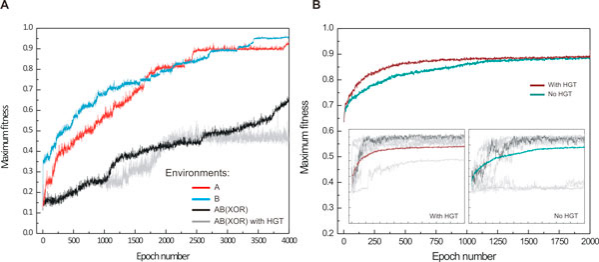
**Fitness trajectories in partial A, B, and full AB (XOR) environments**. (**A**) Evolution of random populations of cells in A, B and XOR environments shown in red, blue, and black respectively. Grey curve shows the averaged fitness trajectory for evolution in a XOR environment with the presence of HGT. Maximum fitness is averaged over 64 simulations for evolution in A and B environments, and over 32 simulations for evolution in a XOR environment. HGT rate here is at 5·10^-5 ^and average fragment size is 4 triplets. (**B**) Evolutionary trajectory under "dual-step" evolution, where population of evolved cells in A and B environments show remarkably fast adaptation to environment AB (64 simulations). HGT confers an additional acceleration of adaptation to new settings. (**B**, inserts) Maximum fitness curves for 8 out of 64 individual simulations with (**B**, left insert) and without (**B**, left insert) HGT are shown in grey. One curve is highlighted with dark grey for clarity.

To assess the effect of HGT in originally non-evolved populations, we placed random populations in all three environments (A, B, and AB) with and without the presence of HGT. In the absence of pre-evolved cells or even partial solutions, the presence of HGT was found not to significantly alter the fitness trajectories of the corresponding populations. As shown in Figure 6A, the fitness trajectories of populations that have been evolved in the AB environment (delayed XOR) under the presence (grey) or absence (black) of HGT are very similar. We expect that in larger population sizes, HGT will be beneficial to integrate beneficial mutations that emerged simultaneously in the populations, and would otherwise compete, although we did not observe such behavior in our populations of relative small size (up to 16,000 cells).

Interestingly, step-wise adaptation and presence of HGT greatly accelerated the rate of evolution. Random populations that were exposed directly to environment AB, required more than 4,000 epochs to evolve the delayed *XOR *function (Figure 6A, black curve). In contrast, populations evolve faster in environments of lower complexity, such as the environments A and B (Figure 6A, red/blue lines). Remarkably, if we sample equal amounts of cells from A and B, and expose the new population in the complex environment AB with all other parameters being equal (size of population, average nutrient concentration, etc.), *XOR *phenotypes of high fitness appear surprisingly fast (Figure 6B). This result complements well recent theoretical predictions [26,27] which suggest that evolution generalizes to new environments through facilitated variation, a process in which genetic changes are channeled in useful phenotypic directions. Our results show that evolution can be accelerated by exposing evolving populations in similar, correlated environments of increasing complexity, and that this effect is even more pronounced in the presence of HGT. Indeed, when HGT is present, the fittest phenotype arises twice as fast as in the absence of HGT events (Figure 6B).

**Figure 7:**
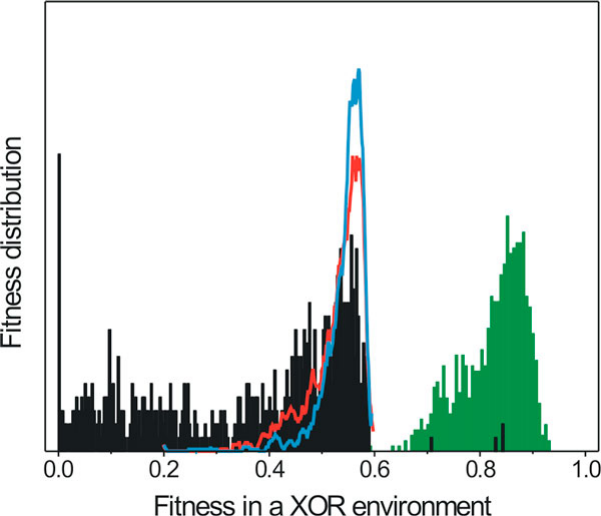
**Fitness probability distribution functions in a XOR environment**. Direct combination of cells evolved in A and B environments does not exhibit a combined *XOR *phenotype. Red and blue lines: cells evolved in A and B environments, respectively. Grey bars: cells constructed by a combination of networks from cells evolved in A and B environments. Majority of combinations have fitness equal or a lower than either of the combined fragments. Few events (4/800) result in a combined fitness higher than 0.6 (1600 cells were randomly selected from 16 populations fully evolved A and B environments, 800 random combined cells were tested for the combined fitness in a XOR environment). Green bars show the fitness distribution of 800 hundred cells collected from the same populations after adaptation to a XOR environment with a presence of HGT.

To further investigate whether this result can be obtained by a simple superposition of the underlying mechanisms, we created a population with organisms whose biological network is the union of networks that belong to organisms evolved in environments A and B, respectively. Contrary to expectations, only 4 out of 800 cells exhibited a fitness increase, while in most cases the network combination resulted to lower fitness when compared to either of the donor cells (Figure 7). Analysis of the mutation/HGT record shows that subsequent fine-tuning mutations after an HGT event are incremental, and often imperative, to its positive fitness effect.

Detailed statistics of the evolution success rate and speed are shown in Tables 1 and 4. During *ab initio *evolution in the AB environment (un-evolved → XOR), only 18 of 32 (56%) experiments were successful and terminated with an evolved *XOR *population (after 4,000 epochs). In contrast, in the case of dual-step adaptation process, where populations where first introduced to environment A or B and then to the AB environment, the success rate was considerably higher to 94% and 85%, with and without HGT, respectively. In addition, HGT accelerates by a factor of 1.7 the emergence of the delayed *XOR *phenotype in {*A*, *B*} mixed populations (measured at 0.75 Pearson Correlation between response protein and nutrient occurrence, which is sufficient and necessary for the XOR I/O characteristic). This HGT effect is less pronounced, however, at the stage of phenotypic refinement (i.e. fitness levels above 0.9) as it is mutations, rather than the integration of new functional fragments (i.e. newly-transferred sets of triplets), that are responsible for fine-tuning of the expression dynamics.

**Table 4:** Probability of the complex *XOR *phenotype emergence in different experimental scenarios.

	*w *> 0.75	***w ***>**0.90**
Unevolved → *XOR*	0.56	0.47
Unevolved → {*A*, *B*} → *XOR*	0.85	0.67
Unevolved → {*A*, *B*} → *XOR ***+ HGT**	0.94	0.68

Organisms with fitness *w *> 0.75 considered to be evolved, further refinement of the phenotype is required to surpass *w *> 0.90 threshold.

### Optimal range for HGT rate and transferred fragment size

We investigated the effect of the fragment size to the fitness trajectory, in the presence of HGT. Towards this goal, we placed initially non-evolved populations in an AB environment that included network fragments taken from already evolved cells. During an HGT event, a cell would have the capacity to probabilistically integrate one of these fragments, as it is in the case of transformation or naked DNA uptake. In the absence of HGT, the averaged final maximum fitness of 32 populations after 4,000 epochs approaches 0.8. Rates of evolution were studied as a function of the average fragment size (Figure 5A) with a fixed HGT rate of 5·10^-5 ^(per cell, per time step). The rate of evolution steadily increases with the increase of the average fragment size transferred by HGT, and saturates at approximately 5 triplets. A closer look at the underlying networks provides the reason behind this saturation limit. In the AB environment the average network size of the evolved organism is 9 triplets, however the average network size that is essential to exhibit the delayed *XOR *phenotype is only 5.5 triplets (see Table 2). Therefore network fragments with 5 triplets or more may contain the full mechanism necessary for a cell to exhibit the *XOR *phenotype, and thus it can be acquired in a single HGT event, if such fragment size is allowed. A similar effect was observed for environments A and B, as well as dynamic AND and OR gates, although the triplet thresholds were lesser due to smaller underlying mechanisms necessary to exhibit the corresponding phenotypes. Hence, the effective fragment size has a saturation limit that depends on the environmental and phenotypic complexity. Noticeably, even HGT of single triplet fragments (orange curve in Figure 5A) significantly accelerates the evolution rate. Even though a single triplet cannot contain a meaningful network, it inherits metabolic triplet regulation, coupling to external signals, basal levels of expression, etc., from the evolved network. Such triplets differ in dynamics from triplets that arise by triplet duplication within the network, and provide a stepping stone for relevant mechanisms to evolve.

Next, we investigated the effect of the HGT rate on the evolutionary trajectory of our populations. For an average fragment size of 4 triplets, we found that there is an optimal dynamic range in which HGT accelerates the emergence of fit phenotypes: rates below 10^-6 ^have little effect on the evolution speed, while rates higher than 10^-3 ^HGT become disruptive to an organism's evolution. Subsequent analysis of the HGT event record and underlying networks revealed that the latter is due to the lack of fixation of advantageous sub-networks/mutations, as the high frequency of HGT events introduces many neutral and deleterious changes that interfere with networks dynamics and operation.

### Phenotypic variability

**Figure 8:**
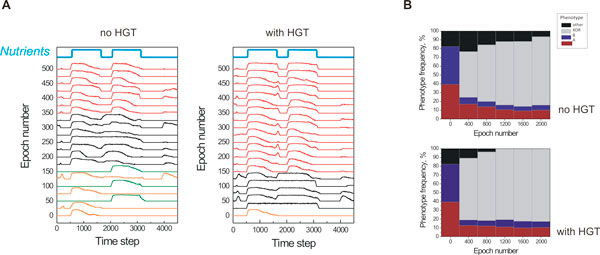
**Metabolic pathway expression and relative phenotypic frequencies during evolution in the AB environment**. (**A**) Emergence of the *XOR *phenotype in a mixed (*A *&*B*) population evolving in the XOR environment. Phenotypes of the fittest organisms of the population are shown for the first 500 epochs of simulation. Without HGT (left panel) cells with initial phenotypes *A *and *B *(orange and green plots, respectively) are the fittest for the first 150 epochs; afterwards cells mutate to intermediate phenotypes (black plots) and finally an *XOR*-like phenotype emerges (red plots). Emergence of the intermediate and final phenotype occurs sooner if networks of type *A *and *B *are combined in one organism by HGT (right panel). Time profile of nutrient abundance is shown for comparison with phenotype profiles on the top of each panel in blue. (**B**) Emergence of an *XOR *phenotype in a population composed as a 1:1 mixture of cells with A and *B *phenotype without (left panel) and with (right panel) HGT in 400 epoch intervals. Frequencies of *A*, *B*, and *XOR *phenotypes are shown in red, blue, and grey respectively; percentage of cells with no distinct phenotype is shown in black. Statistics is collected over 64 experiments.

In the presence of HGT and in combined populations, the *XOR *phenotype propagates through the population at a significantly faster rate (750 *vs*. 1400 epochs, Figure 6B). In the absence of HGT, the fittest phenotype in the population alternates between those of environment A and B (orange and green curves, Figure 8A) until new "hybrid" phenotypes emerge (black curves) that eventually give rise to highly fit, *XOR *phenotypes (red curves). The presence of HGT clearly leads to a faster emergence of fit phenotypes (30 epochs *vs*. 150 epochs). In addition, we looked at how the rate of evolution scales as a function of the population size. It is believed that evolution speed increases linearly with population size N for small populations, and with ln(N) for intermediate population sizes [28], while it approaches a saturation limit for large populations (> 10^9^) [29]. In our simulations, we observed a linear dependence of evolution rate to population size in accordance to theoretical predictions (Additional file 1).

To examine the phenotypic variability and substitution rates in our experiments, we created a temporal profile of relative phenotypic frequencies during the course of 64 simulations. All initial organisms in the population that exhibited phenotype *A *and *B*, along with their offspring, were found to be replaced within 50 epochs by the evolved *AB (XOR) *phenotype, once the latter arises. However, both phenotypes A and B persisted until the end of the simulation runs and at relatively high percentages (10% to 18% of total population size, Figure 8B). Analysis of the fossil mutation record of these cells revealed that this is due to reversing mutations of the AB phenotype to either A or B, which is what we would expect from mutation-selection balance theory. Interestingly though, we observed multiple solutions (i.e. methods of regulation and pathways) for the same phenotype that co-existed in the population, in agreement with the quasi-species theory [30], which provides its first computational example in the context of gene regulatory and biochemical networks.

### Distribution of fitness effect of mutation and HGT events

Mutations and LGT events differ in magnitude and direction when it comes to their fitness effect. Traditionally, models rely on theoretical or experimentally constructed distributions of fitness effect (DFE) when introducing mutations in a population. These distributions have been measured experimentally for viruses and bacteria [31-37] and have also been obtained theoretically (e.g. [38] and references therein). Briefly, experimental methods rely on analysis of genomic data, where the assumption is that mutation fixation probability is analogous to the benefit it confers [39], or engineered single-nucleotide substitutions in bacteria [31,32] and viruses [33-36] with small genome size. In general, it is assumed that most mutations have a neutral or nearly neutral effect and the vast majority of mutations have a negative fitness effect [38]. In bacteriophage F1, 20% of single point mutations were found to be lethal, while the mean fitness decrease was around 11% [34]. In *E. coli*, the average effect of spontaneous deleterious mutations and random insertions is less than 1% and 3%, respectively [31,32].

Theoretical approximations of the DFE are usually based on the concept that most mutations are "nearly neutral" as introduced by Kimura [40]. Following this theory, the shape of DFE is assumed to peak close to zero and skew towards deleterious mutations. Another common framework is the Fisher's adaptive landscape model [41,42], although it is considered to have a heuristic, and not quantitative value [43] due to its simplifying assumptions. In recent studies, application of the extreme value theory resulted to an exponential distribution of beneficiary mutations [44], although computational studies of RNA evolution found to be better described by a Gumbel distribution with an exponential right shoulder only if 99% of nearly neutral observations are discarded [45]. One simplifying assumption in both theoretical and experimental studies is that the shape of DFE remains the same during evolution, which it contradicts our findings. In addition, both experimental and theoretical work is focused on static environments and in already evolved organisms. Despite the large number of studies on the mutation DFE, the fitness distribution for Horizontal Gene Transfer events remains unknown.

**Figure 9:**
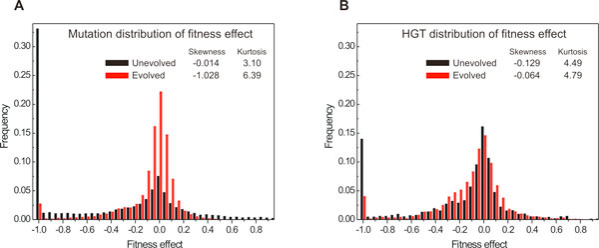
**Distribution of fitness effect (DFE) in unevolved (black) and evolved (red) populations**. Effect of (**A**) mutation and (**B**) HGT events. As populations evolve, frequency of neutral mutations increases, but frequency of neutral HGT events stays almost unaffected; in both cases frequency of lethal events is decreased in the evolved populations. Skewness and kurtosis of the distributions is shown on the top; more details can be found in Table 3. For each of four plots, statistics is collected over 8 populations evolved for 100 epochs in a XOR environment.

Here, we use our *in silico *simulation framework to investigate the shape and changes in the DFE for both mutations and HGT. Since each organism has its own regulatory network that results to a distinct phenotypic behavior, we are able to calculate fitness before and immediately after any HGT event (correlation between expression levels of the response pathway and nutrient presence). This allows us to profile the shape of DFE along the evolutionary trajectory and to account for genetic drift, which can be a significant force in small populations. Figure 9 shows the DFE for eight evolved and non-evolved populations in the AB environment (delayed XOR) over the course of 100 epochs. Non-evolved populations were randomly generated, while evolved populations were constructed from the high fitness cells (*w *> 0.75) sampled from populations that had evolved in an XOR environment for 8,000 epochs. In both mutation and HGT DFEs, there is a profound decrease in the number of lethal events (i.e. fitness effect equal to -1) in evolved populations versus the non-evolved populations, a clear indication that the latter become robust to mutations. Table 3 provides a synopsis of the DFE parameters of the DFE for viable mutations and HGT events (fitness effect > -0.8), and shows that the resulting mutation DFE is in good agreement to the effect of measured single point mutations in viruses and *E. coli*. Furthermore, we observe a decrease in variance and increase in kurtosis (sharpness) of the DFE as populations evolve, both for HGT and mutations, although the effect is more profound in the latter case. As population evolves, mutation DFE becomes more skewed towards negative fitness effects, which is to be expected as most mutations in an evolved organism result in a decreased fitness. Interestingly, the DFE of HGT events becomes more symmetric (skewness closer to zero) in evolved populations, as the probability for HGT to transfer a beneficial or disrupting fragment increases (the first because of the availability of beneficial sub-networks, the second because of the high ratio of already fine-tuned cells in the population that can be disrupted by a HGT event).

**Figure 10:**
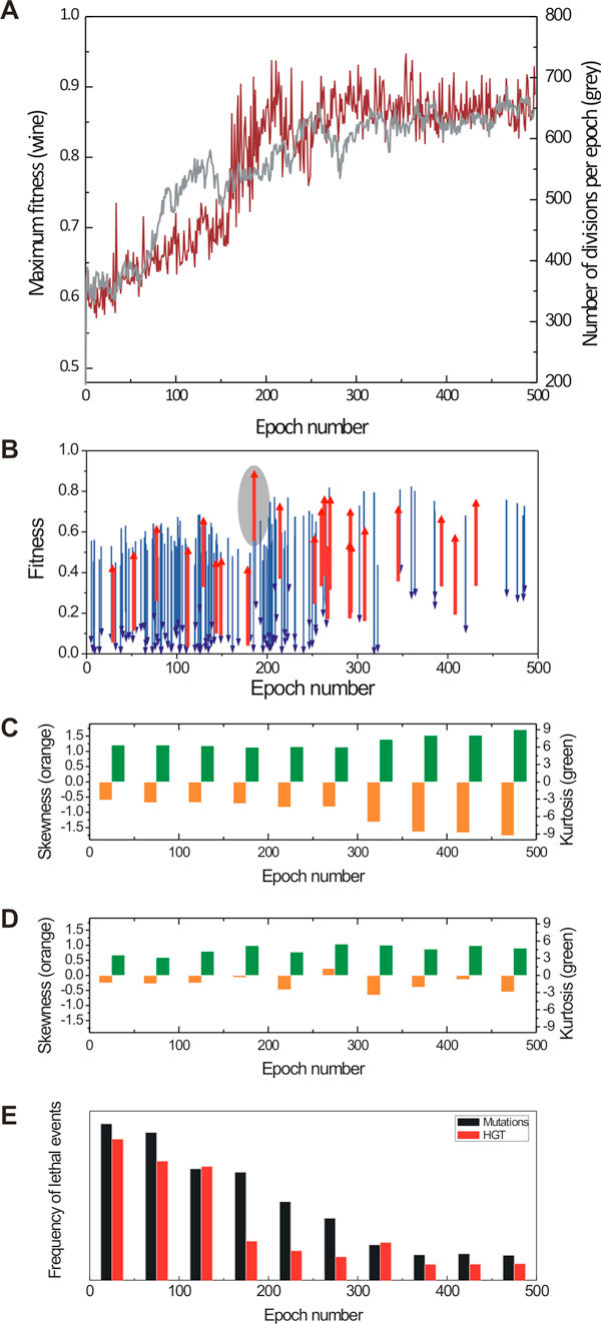
**A population composed of cells evolved in partial A and B environments evolving in a combined XOR environment**. (**A**) maximum fitness trajectory (wine) and number of divisions per epoch (grey); additional details for this evolutional trajectory are shown in the following panels: (**B**) HGT events with strong positive (increase in fitness Δ*w *> +0.3) and very strong negative (decrease in fitness Δ*w *< -0.4) fitness effects are shown with red and blue arrows, respectively; origins and heads of arrows represent fitness before and after HGT event, respectively; red arrow highlighted with the grey oval represents the HGT event, which resulted in the emergence of a fully evolved cell in this population (this event is described in detail in Fig. 4) (**C**) skewness and kurtosis of DFE for non-lethal *mutation events *calculated for every 50 epochs; (**D**) same as above, DFE of non-lethal *HGT events*; (**E**) frequency of lethal (deleterious) mutation and HGT events as a function of evolutionary time.

To gain more insight on the DFE changes, we traced the fitness effect of mutations and HGT events along a single evolutionary trajectory. Figure 10 shows the 500-epoch fitness trajectory in the AB environment of a population composed of cells that had been pre-evolved in either A or B, and subsequently mixed. Initial maximum fitness in a pre-evolved population is above 0.5 and reaches 0.85 after 200 epochs. While growth (i.e. number of divisions, grey line) is nearly exponential for the first 100 epochs, it saturates due to nutrient limitation. The HGT DFE skewness and kurtosis do not change significantly over time, while the DFE for mutations steadily becomes sharper and skewed to the left (Figure 10C and 10D). In addition, we observe a steady decrease of lethal mutation and HGT events during the course of evolution (Figure 10E), with a sharp decrease of lethal HGT events around 175 epochs, which correlates with the emergence of the highly fit phenotype in the population.

### Network organization

The complete gene regulatory and biochemical network of an evolved cell is usually too complex to analyze, while many of its connections are not relevant to the observed phenotype. To address this issue, we employed a reduction algorithm (see Methods) to extract the "minimal" network that encompasses only essential connections. As shown in Table 2 average fitness of reduced minimal networks is at least 95% of the full network's fitness, however the average number of regulatory edges is significantly reduced: from 338 to 14.1 and from 335 to 10.6 with and without HGT, respectively. Presence of HGT events results in larger networks that are considerably more sparse (0.39 *vs*. 0.22), but with the same average sparsity and reduced network size difference when it comes to their minimal counterparts.

To elucidate how HGT confers a fitness advantage during evolution, we dissect the gene regulatory and biochemical network of a cell before and after an HGT event. Figure 4 depicts an HGT event that integrates a network fragment from a cell with phenotype B into a cell with phenotype A during evolution in the AB environment. This particular event had occurred at epoch 185 of the evolutional trajectory shown in Figure 10B by a highlighted red arrow. The resulting cell has a high fitness *w *= 0.84 in the AB environment (XOR) which is unusually high for the given timeframe. The expression profile of the donor, acceptor and final cell is depicted in Figure 4A. As shown, the acceptor and donor cells respond well to the presence of the first and second occurrence of nutrients, respectively. The HGT event introduces a fragment that incorporates vital components of the mechanism employed by the donor cell to infer the presence of the second nutrient pulse, which does so by processing information carried by signal S_1_. The resulting cell is able to metabolize the nutrients during both pulses, and its response expression profile correlates well with the nutrient presence. Subsequent mutations result in the fine-tuning of the timing for the expression of the response pathway, which then correlates better to the second nutrient pulse (data not shown).

Figure 4B shows the minimal network of the resulting cell, with the minimal network of the acceptor cell with A phenotype (*Delayed *(*s_1 _*AND NOT(*s_2_*))) being in a solid yellow background. Activation of the metabolic (response) pathway RP_0 _of triplet T0 is regulated by the protein from the triplet T2: signal S_1 _activates transcription of T3, protein T3 controls translation of T1, which in turn regulates the transcription of T2. The delay is introduced by activation of the protein modification in T2 by the same signal S_1_, which decreases concentration of un-modified protein T2 while signal S_1 _is present. Once signal S_1 _is off, modified proteins T2 gradually return to the un-modified state and metabolic pathway RP_0 _becomes activated. Signal S_2 _regulates protein modification in T3, which blocks metabolic pathway RP_0 _expression when both signals S_1 _and S_2 _are present, and therefore satisfies NOT(*s_2_*) part of the environment. The HGT event introduces three extra triplets that add the additional functionality as follows: signal S_2 _activates transcription of T5, which in turn activates the metabolic pathway RP_0_. Signal S_1 _activates transcription of T4 and T6, which inhibit translation of T5. Triplet T6 has parameters almost identical to T4, since it was created by triplet duplication earlier during the evolution. However in this cell, both T4 and T6 are present in the minimal network to maintain a required level of inhibition.

## Conclusions

Horizontal gene transfer is a phenomenon that inarguably affects the evolution and emergence of complex traits. To what extent it does so, its impact to the underlying networks and population dynamics, however, is still to be determined. Our multi-scale models and simulations allow, for the first time, to address questions related to HGT that transcend three levels of organization (microbial populations, organism, and regulatory networks), shedding light on its effect during evolution and providing concrete examples of network integration and operation. Our results show that the effect of HGT is very much dependent on the environmental context which includes, but is not limited to, the genotypic and phenotypic variability of the existing populations. The observation that HGT can accelerate the rate of evolution of heterogeneous populations is intriguing: can we experimentally manipulate the rate of evolution by sequentially exposing a population to fluctuating environments in the presence of HGT? What are the principles and rules that optimize this effect? Our computational results show that adaptation to correlated environments of increasing complexity can accelerate the rate of evolution, especially in the presence of HGT, a hypothesis that remains to be tested in a laboratory setting. HGT events show a distinct spectrum in respect to their fitness effect. The HGT-derived fitness effect distribution was found to increase its symmetry during evolution, in contrast to the fitness effect distribution of mutations, which becomes more skewed towards deleterious mutations. Our distributions of mutation effects are in complete agreement with experimental observations so far, while our predictions regarding the reported DFE bias remain to be tested experimentally.

There are many future directions to explore in order to increase the scope of the methods presented here. In respect to the models, the current framework will benefit from the addition of a spatial component, since in the current setting we assumed a well-mixed, homogeneous environment which doesn't allow the investigation of individual HGT mechanisms (transduction, conjugation, transformation), as their effect varies greatly with the spatial landscape of the environment and population structure. Moreover, the same simulations can be performed with a model of adaptively growing populations which are only nutrient-limited instead of having a fixed-size population, although the latter is easier to analyze and balance in simulations. A larger population size is always desired as small effective population size renders results prone to undesired artifacts, such as random drift, although this is less of a concern here as we can *a posteriori *analyze the effect of every and each mutation event during the evolution. The proposed framework can serve as a predictive and testing tool for evolutionary hypotheses that would be difficult to evaluate experimentally, while at the same time can drive laboratory experimentation by providing scenarios that are likely to occur in specific environmental contexts.

## List of abbreviations used

DFE: distribution of fitness effect; HGT: horizontal gene transfer.

## Competing interests

The authors declare that they have no competing interests.

## Authors' contributions

Corresponding author IT conceived the project; VM wrote the code and performed the experiments; VM and IT designed the methods, analyzed the data and wrote the manuscript.

## Supplementary Material

Additional file 1 **Evolution rate as a function of the population size**. Rate is calculated as an average slope of the maximum fitness increase averaged over 8 independent experiments for each population size of 256, 512, 1024, 2048, 3072 and 4096. Initial random populations evolved in the XOR environment until the maximum fitness is stabilized. Click here for file

Additional file 2 **HGT fragment transfer probability and genome integration**. (**A**) Probability density function profile used to select HGT fragment sizes. (**B**) Incorporation of the HGT-transferred fragment (triplets T_N+1 _to T_N+k_) in the regulatory matrix, where only the response pathway (triplet T_0_) regulation is conserved. The newly acquired fragment can over time rewire and couple to other nodes in the network. Simulations where the imported fragment was randomly rewired to the host genome yielded similar results. Click here for file
