# Supplementary figures and images for: Horizontal gene transfer dynamics and distribution of fitness effects during microbial in silico evolution
Source: BMC Bioinformatics. 2012 Jun 25;13(Suppl 10):S13. doi: 10.1186/1471-2105-13-S10-S13 (PMC3382434; doi:10.1186/1471-2105-13-S10-S13)

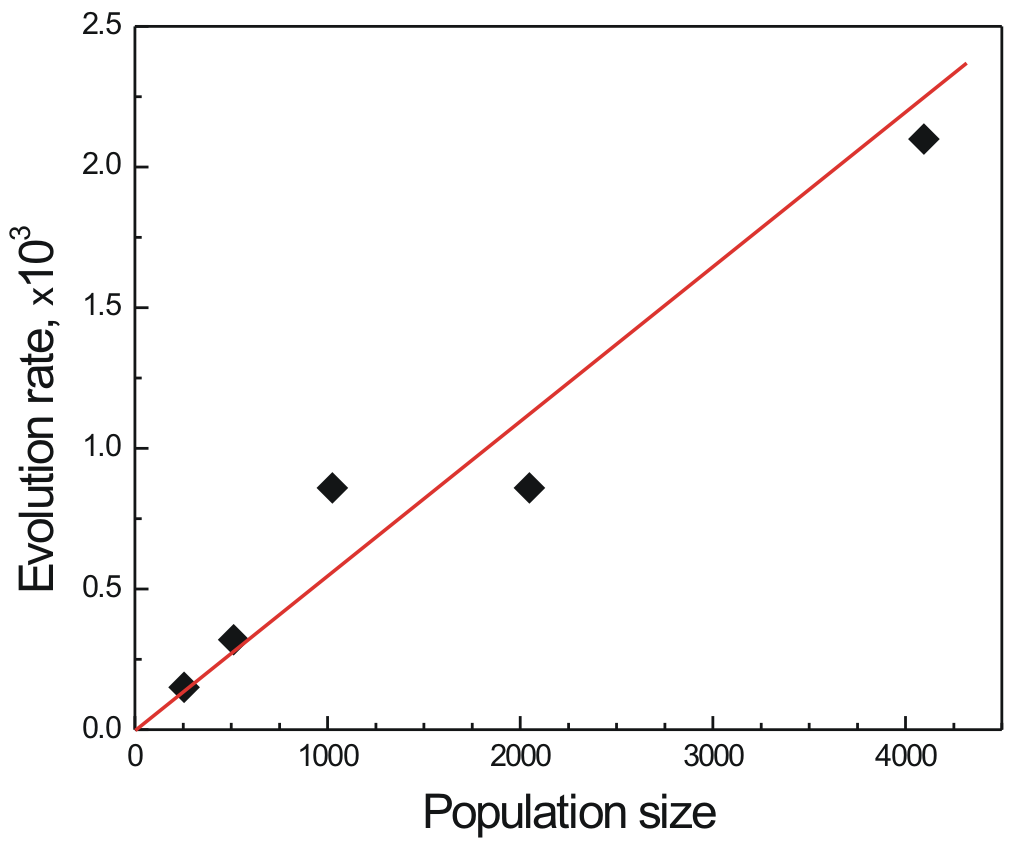

Supplement: Additional file 1 — Evolution rate as a function of the population size. Rate is calculated as an average slope of the maximum fitness increase averaged over 8 independent experiments for each population size of 256, 512, 1024, 2048, 3072 and 4096. Initial random populations evolved in the XOR environment until the maximum fitness is stabilized. [file 1471-2105-13-S10-S13-S1.tif]

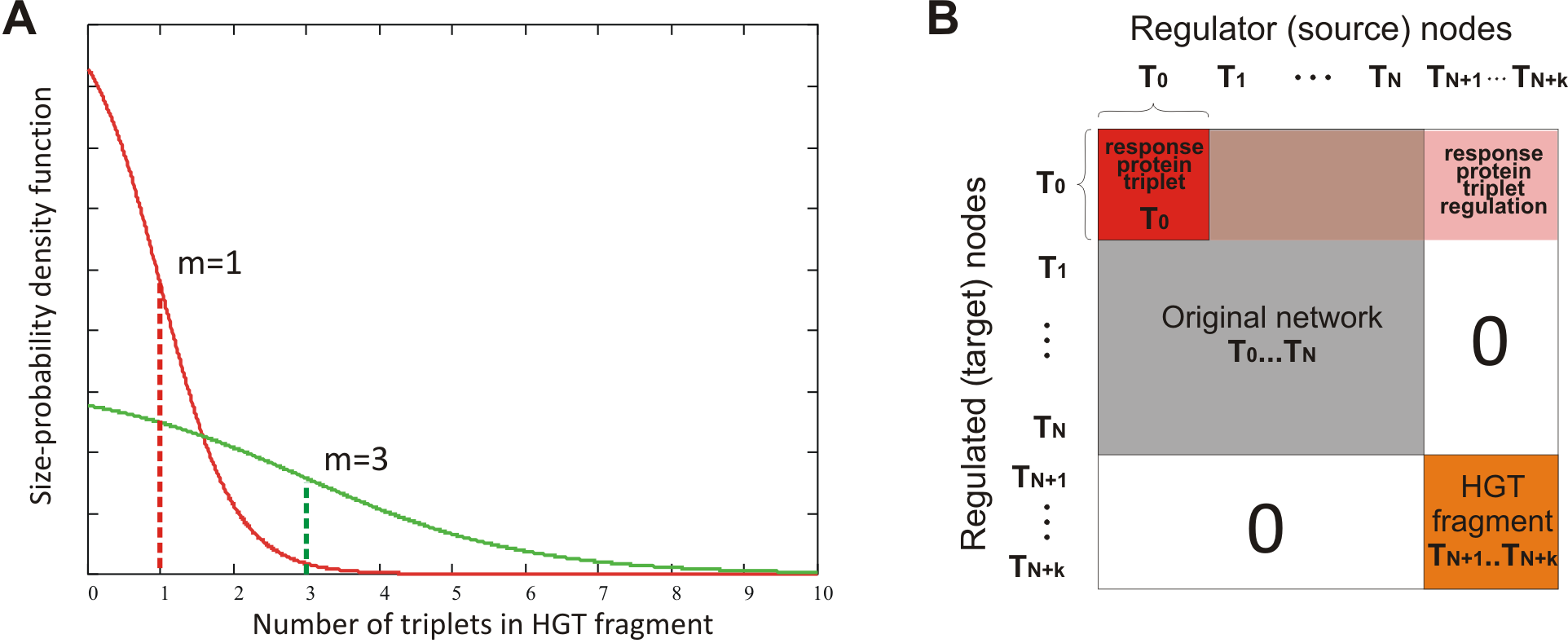

Supplement: Additional file 2 — HGT fragment transfer probability and genome integration. (A) Probability density function profile used to select HGT fragment sizes. (B) Incorporation of the HGT-transferred fragment (triplets TN+1 to TN+k) in the regulatory matrix, where only the response pathway (triplet T0) regulation is conserved. The newly acquired fragment can over time rewire and couple to other nodes in the network. Simulations where the imported fragment was randomly rewired to the host genome yielded similar results. [file 1471-2105-13-S10-S13-S2.tif]
